# Conversion from Minimally Invasive Surgical Approaches to Open Surgery Among Patients with Endometrial Cancer in the SGO Clinical Outcomes Registry

**DOI:** 10.1245/s10434-025-16949-y

**Published:** 2025-02-21

**Authors:** Abdelrahman Yousif, Julie Ngo, Deena Abdel-Gadir, Rodney P. Rocconi, Patrick Timmins, Jason Lachance, J. Michael Straughn, Summer Dewdney, Jenny Lachance, Benjamin Mize, IIana Chefetz

**Affiliations:** 1 OBGYN Department, Texas Tech University Health Sciences Center, El Paso, TX USA; 2 Henry Ford Hospital Family Medicine Residency, Detroit, MI USA; 3 College of Human Medicine, Michigan State University, Flint, MI USA; 4 University of Mississippi Medical Center, Jackson, MS USA; 5 Women’s Cancer Care Associates, Albany, NY USA; 6 Maine Health, Scarborough, ME USA; 7 University of Alabama at Birmingham, Birmingham, AL USA; 8 Rush University Medical Center, Chicago, IL USA; 9 Research Department, Hurley Medical Center, Michigan State University, Flint, MI USA; 10 Department of Gynecologic Oncology, Karmanos Cancer Institute, Wayne State University, Detroit, MI USA; 11 Department of Biomedical Sciences, Mercer University School of Medicine, Macon, GA USA

**Keywords:** Endometrial cancer, Minimally invasive surgery, Conversion, Open surgery, Prior abdominal surgery, Uterine size, Blood transfusion, Risk factors, Hysterectomy, Chemoresistance, Anemia

## Abstract

**Background:**

Endometrial cancer (EC) ranks as the most common gynecologic malignancy in the USA. While minimally invasive surgical (MIS) techniques have revolutionized EC management, conversion to laparotomy remains a concern due to the loss of laparoscopic benefits such as fewer surgical site infections and shorter hospital stays with reported rates varying widely. Factors influencing this conversion, including patient characteristics and tumor attributes, have not been fully understood. Our study aims to provide a framework for identifying patients at higher risk of conversion, thereby helping to inform surgical decision-making and patient counseling Addressing this gap, our study employs a national registry to analyze patient- and tumor-related factors associated with the transition from MIS to open surgery in EC.

**Patients and Methods:**

We queried the SGO Clinical Outcomes Registry (COR) to identify all patients with EC who underwent surgical management. The COR indeed validated clinical data from 29 sites collected between 2014 and 2018. The primary outcome was to assess the conversion rate from MIS to open surgery. Descriptive statistics using means with standard deviations or frequency with percentages were used. Chi-squared analysis was used to examine the bivariate relationship between group status and the subjects’ demographic and clinical variables.

**Results:**

A total of 3.4% (135/4028) of patients underwent conversion from MIS to open surgery. Demographic characteristics were balanced between the groups. Conversion was more prevalent in patients with obesity (29%) and morbid obesity (37%) than in patients who are underweight (2%), normal weight (16%), and overweight (16%). Similarly, conversion was more prevalent in patients with prior abdominal surgery (63% versus 52%; *P* = 0.001). Endometrioid (EC) predominated (59%) in the converted group, with higher-than-expected non-endometrioid rates (serous carcinoma 16%, clear cell carcinoma 4%, carcinosarcoma 5%, mixed histology 12%; all *P* < 0.01). Advanced International Federation of Gynecology and Obstetrics (FIGO) stages were more common in patients who converted to open surgery (stage II: 5%, stage III: 25%, stage IV: 9%; all *P* < 0.001). Type II (24%) and type III (5%) hysterectomies were more frequent in patients who converted to open (*P* < 0.001). Logistic regression indicated body mass index (BMI), prior surgery, FIGO stage, histology, and hysterectomy type affected conversion (*P* < 0.001), explaining 12.3% of the variance in the conversion outcome. Indications for conversion included uterine size, adhesions, and disease extent.

**Conclusions:**

The adoption of MIS has become increasingly common standard of care for managing EC, attributed to enhanced perioperative outcomes. Factors associated with conversion such as uterine size, prior abdominal surgeries, surgical complexity, disease extent, and histologic types can affect the surgeon’s choice. Ultimately, a personalized surgical approach, tailored to individual patient attributes, remains pivotal for optimizing outcomes in EC management.

Endometrial cancer (EC) is the most common gynecologic malignancy in the USA.^1^ Traditionally, the management for most patients with EC was total abdominal hysterectomy performed via a vertical midline incision. With advances in technology, minimally invasive surgical (MIS) approaches have become widely adopted by gynecologic oncologists for management of EC.^1^ MIS approaches have been associated with improved patient outcomes, including reduced intraoperative blood loss, shorter operating and anesthesia times, quicker recovery, decreased risk of wound infection, shorter hospital stays, and less postoperative pain.^2^ Large multi-institutional randomized controlled trials (RCT) have demonstrated either the superiority or noninferiority of MIS approaches compared with laparotomy with respect to surgical and oncological outcomes. However, not all MIS procedures can be completed as planned, and conversion to open surgery has been associated in some reports with an increase in adverse outcomes for patients.^3^ The literature reports varying rates of conversion, likely due to heterogeneity in patient cohorts, surgeon qualification, and tumor characteristics. Conversion rates in the literature range from 5.5 to 5.8% and up to 25% in the Gynecologic Oncology Group Study GOG LAP2 trial.^4–6^ A better understanding of these factors could help surgeons make more well-informed decisions when planning surgery with MIS techniques for EC. Factors such as large uterine size, patient age, and body mass index (BMI) have been theorized as reasons for conversion to open surgery in previous studies.^7^

Our study aims to use the Society of Gynecological Oncology (SGO) Clinical Outcomes Registry (COR) to investigate patient- and tumor-related factors associated with conversion from MIS to open surgery in patients with EC.^8^ Due to registry limitations, detailed surgical histories and specific surgeon-related factors are not available.

## Patients and Methods

The SGO undertook an initiative to collect clinical outcomes data relating to gynecologic cancers in 2013 by creating a registry. The registry was activated in January 2014 and accrued patients through January 2018. Approximately 29 individual sites collected the data with standardized forms. At the top 9 accruing sites, 18 validation data points were audited. Each site audited 10% of randomly selected charts for these data points. A total of eight of nine sites had a 100% completion rate for data entry.

Statistical analysis for validation of the SGO registry found for 613 EC cases an average 88.4% agreement and kappa coefficients ranging from 0.3 to 0.85.^8^ This was an Institutional Review Board (IRB)-exempt cross-sectional analysis of clinical and surgical outcomes among patients with EC in the SGO COR. Our study included all patients with EC who underwent surgical management. Patients were stratified by the conversion from MIS to open surgery outcome. All data were analyzed descriptively using means with standard deviations or frequency with percentages. Chi-squared analysis was used to examine the bivariate relationship between group status and the subjects’ demographic and clinical variables. The alpha level for this study was set at 0.05.

A backward logistic regression model was used to identify possible predictors of conversion. The following variables included BMI, prior abdominal surgery, International Federation of Gynecology and Obstetrics (FIGO) stage, histology types, operation types, and uterine weight group. At each step, variables continued in the model if they met the criteria of a *P*-value threshold of 0.10 or less.

## Results

**Fig. 1:**
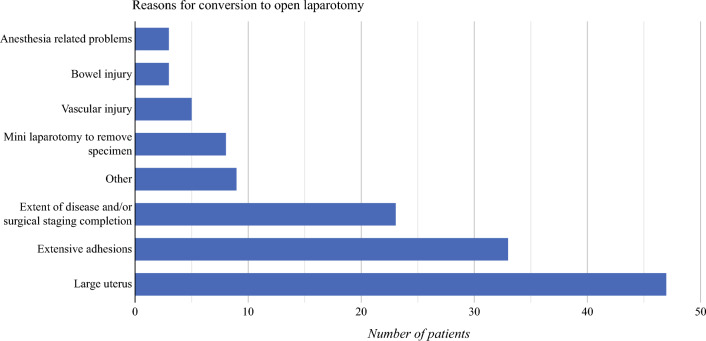
Indications for conversion to open surgery; other includes significant visceral adiposity (1), obturator nerve injury (1), bladder injury (1), not reported (3), unknown (3)

A total of 135 (3.4%, 135/4028) patients underwent conversion from MIS to laparotomy for EC surgical staging. Figure 1 shows indications of conversion to open surgery with uterine size and presence of extensive adhesions. In addition, extent of disease/surgical staging completion was found to be the most frequent indication for conversion. No significant difference was found by age or race. The majority of patients with EC who underwent conversion from MIS to laparotomy were either obese (29%) or morbidly obese (37%), (*P* = 0.04) compared with the MIS group.

No differences were found between the groups when comparing the most common medical comorbidities, hypertension, diabetes, and hypothyroidism. Prior abdominal surgery was associated with conversion compared with 63% versus 52%, respectively (*P* = 0.001) (Table 1).

**Table 1:** Patients’ demographics, medical comorbidities, and history of prior abdominal surgeries stratified by the conversion to open surgery event

	Converted		MIS		*P* value
	*N*	%	*N*	%	
Total number	135		3893		
Race					
Caucasian	113	84	3420	88	0.187
African American	13	10	233	6	0.079
Hispanic	5	4	92	2	0.515
Asian	2	2	30	1	0.357
Unknown	1	1	55	1	0.515
BMI					0.040
Underweight	2	2	18	1	
Normal	22	16	458	12	
Overweight	22	16	721	19	
Obese	39	29	1494	38	
Morbid obese	50	37	1202	31	
Medical conditions					
Hypertension	82	61	2129	55	0.165
Diabetes	39	30	998	26	0.302
Hypothyroidism	19	14	719	19	0.194
Atrial fibrillation	11	8	213	6	0.182
Congestive heart failure	1	1	42	1	0.707
Myocardial Infarction within 6 months	2	2	16	0	0.067
Chronic obstructive pulmonary disease	3	2	145	4	0.362
Prior abdominal surgery					< 0.001
Yes	83	63	1993	52	
No	48	37	1859	48	
Age (mean)	64.63 ± 10.62		63.91 ± 10.43		0.435

Underweight 12.7–18.4, normal 18.5–24.9, overweight 25–29, obesity 30–39.9, morbid obesity ≥ 40

Endometrioid EC is the most common histological subtype. There was a higher percentage of endometroid EC in the MIS group (79%) compared with the converted group (59%), respectively (*P* < 0.001). Non-endometroid histology was more frequent among the converted group compared with the MIS group (serous carcinoma at 16% versus 8% (*P* = 0.02), clear cell carcinoma at 4% versus 2% (*P* = 0.006), carcinosarcoma at 5% versus 3% (*P* = 0.229), and mixed histology 12% versus 5% (*P* < 0.001)). FIGO stage I was the most commonly reported stage with 56% among the converted group and 76% among the MIS group (*P* < 0.001). A higher percentage of patients in the converted group had advanced FIGO stages compared with MIS patients (*P* < 0.001); 5% versus 4% for stage II, 25% versus 8% for stage III, and 9% versus 2.5% for stage IV (Table 2).

**Table 2:** Histological subtypes and FIGO stages stratified by the conversion event

	Converted		MIS		*P* value
	*N*	%	*N*	%	
Histology					
Endometrioid	79	59	3083	79	< 0.001
Serous	21	16	311	8	0.002
Mixed	16	12	189	5	< 0.001
Carcinosarcoma	7	5	128	3	0.229
Clear	6	4	57	2	0.006
Leiomyosarcoma	2	2	2	0	< 0.001
FIGO stage					< 0.001
IA	56	42	2487	64	
IB	19	14	621	16	
II	7	5	174	5	
IIIA	14	10	146	4	
IIIB	3	2	40	1	
IIIC1	10	7	190	5	
IIIC2	9	7	55	1	
IVA	3	2	21	1	
IVB	10	7	78	2	
Tumor grade					< 0.001
Grade 1	44	33	2048	53	
Grade 2	29	22	912	23	
Grade 3	57	42	832	21	

On the basis of our findings, among patients who underwent lymphadenectomy, the conversion rate was the highest among patients undergoing para-aortic lymphadenectomy compared with pelvic and sentinel lymphadenectomy, at 4%, 3%, and 2%, respectively. Pelvic sentinel lymph node biopsies were more often performed in patients who did not experience conversion (33% versus 17%; *P* = 0.002) compared with the converted group. There were no statistically significant differences between groups concerning pelvic or para-aortic lymphadenectomy rates. Type I hysterectomies were the most common hysterectomy type in both groups. More patients who experienced a conversion to open surgery underwent type II or type III hysterectomy compared with the MIS group; 24% versus 14%, respectively, for type II (*P* = 0.001) and 5% versus 2%, respectively, for type III (*P* < 0.001). Conversion rate was found to be the highest among patients who underwent type III hysterectomy (8% compared with 6% for type II hysterectomy and 3% for type I hysterectomy; Table 3).

**Table 3:** Different surgical procedures performed during surgical staging and initial surgical approach stratified by the conversion outcome

	Conversion rate	Converted		MIS		*P*-value
	%	*N*	%	*N*	%	
Sentinel lymphadenectomy						< 0.001
Yes	2	24	18	1255	33	
No	4	109	82	2599	67	
Pelvic lymphadenectomy						0.157
Yes	3	81	60	2561	66	
No	4	53	40	1300	34	
Para-aortic lymphadenectomy						0.119
Yes	4	41	31	951	25	
No	3	93	69	2903	75	
Laparoscopy	4	47	35	1128	29	0.142
Robotic assisted laparoscopic hysterectomy	3	89	66	2899	75	0.026
Type of hysterectomy performed						
Type I	3	92	68	3161	81	< 0.001
Type II	6	33	24	530	14	< 0.001
Type III	8	7	5	86	2	0.024

Type I: simple hysterectomy, Type II: modified radical hysterectomy, Type III: radical hysterectomy

We next performed a backward logistic regression to examine the effects of BMI, prior abdominal surgery, FIGO stage, histology types, operation types, and uterine weight on conversion status (Table 4). The model was statistically significant (*P* < 0.001), with 96.6% of cases correctly classified. Our model consists of preoperative and intraoperative factors that provide valuable information for both patients and surgeons to address better and discuss the risk of conversion. See Table 4 for information on statistically significant variables.

**Table 4:** Significant predictors of conversion with adjusted odds ratio and 95% confidence intervals

	*P* value	Adjusted* odds ratio	95% CI for OR	
Prior abdominal surgery	< 0.001	1.993	1.331	2.954
FIGO stage	< 0.001	1.171	1.082	1.268
Histology/serous	0.011	2.092	1.189	3.683
Histology/clear cell	0.003	4.064	1.597	10.341
Histology/leiomyosarcoma	0.012	17.943	1.867	172.459
Histology/mixed	0.023	2.065	1.104	3.863
Operation/hysterectomy Type I	0.008	0.562	0.368	0.858
Uterine group size	< 0.001	3.746	2.486	5.643

*Adjusted for age, BMI, and medical comorbidities

Uterine size was classified into two groups with those 40–249 grams coded as “0” and 250 or more grams coded as “1”

## Discussion

Since the publication of LAP2, MIS has been widely adopted for the surgical management of EC, leading to better operative outcomes and reduced surgical morbidity. Consistent with some previous studies, our study reported a low conversion rate.^7,9^ However, the GOG LAP2 trial from 2009 reported a higher conversion rate of 25%, likely the result of a requirement for full pelvic.

In our study, parameters such as race and major medical comorbidities did not show any significant differences between groups, but patients requiring conversion to open surgery did have a higher incidence of obesity, morbid obesity, prior abdominal surgeries, non-endometrioid histology, and higher FIGO stage. The need for type II or type III hysterectomy also exhibited a stronger association with conversion to open surgery.

Patient selection and preoperative surgical planning play a critical role in determining the appropriate surgical approach in clinical practice. In a study conducted by Matsu et al., it was found that the advanced age of the patient and uterine size were the most significant predictive factors for conversion to open surgery.^10^ Specifically, the risk of conversion was found to be three times higher in patients with a uterine size of 250 mg or higher.^10^ Similarly, in our present study, a large uterus was identified as the most common indication for conversion to open surgery, followed by adhesive disease and extent of disease, which were the top three indications for conversion. In addition, our findings revealed that a higher uterine weight was significantly associated with an increased risk of conversion to open surgery, even after adjusting for other relevant variables. This highlights the importance of considering uterine weight as a significant factor in the preoperative surgical planning process. Additionally, the surgical history of patients was found to be an integral part of preoperative planning, as it can contribute to the presence of extensive adhesions, making the surgery more technically challenging and ultimately leading to conversion to open surgery. Notably, our data showed a slightly higher, yet statistically significant, percentage of patients who underwent conversion to open surgery had a history of previous abdominal surgeries.

Previous research has investigated the utilization and outcomes of MIS in patients with high-risk histologic subtypes and advanced FIGO stage of EC. To address the existing uncertainty surrounding the use of MIS in patients with high-risk histologic EC, a recent meta-analysis conducted by Kim et al., which included nine retrospective studies, examined the use of MIS in this cohort. This meta-analysis demonstrated that MIS was not associated with increased mortality or recurrence risk compared with open surgery. The conversion rate from MIS to open surgery varied among the included studies, ranging from 2 to 9.9%.^11^ Similar results were reported in a systematic review by Scaletta et al., which indicated that MIS is considered to be a safe option for managing patients with high-risk histologic EC, with comparable oncologic outcomes to open surgery.^12^ Furthermore, our results confirmed that high-risk histologic subtypes, such as uterine serous carcinoma (USC) and clear cell carcinoma, are independently associated with conversion to open surgery.

Numerous studies have provided evidence supporting the safety and feasibility of MIS in patients with advanced FIGO stage endometrial cancer (stage ≥ II).^13–15^ In Tanaka et al.’s study, 12 patients were excluded due to conversion events, with advanced-stage disease accounting for 6 out of the 12 conversions.^15^ Our findings corroborate these findings, revealing a significant association between the advanced FIGO stage and the increased likelihood of conversion from MIS to open surgery. Specifically, our results indicate that 25% of patients who experienced a conversion event had stage III disease, compared with only 8% of those who completed their primary surgery minimally invasively. Furthermore, 9% of patients with conversion complications had stage IV disease, compared with only 2.5% of those who completed their surgeries minimally invasively (*P* < 0.005).

### Limitations

This study has several limitations. First, the study’s retrospective design inherently carries limitations associated with this type of research approach. Although efforts were made to collect data from a large sample size and trained personnel were involved in data collection, the absence of complete operative reports, preoperative imaging, or patients’ records may have limited the extent of data extraction. These data are collected from high-volume and top-performing surgical centers, and this could have contributed to the low conversion rate among the study cohort. Moreover, the cohort studied represents a group of patients with EC managed by surgeons with specialized training in gynecologic oncology, which may not be generalizable to other geographical/clinical care settings. The study’s retrospective nature may introduce selection biases and limits causal inferences.

## Conclusions

The use of MIS has become the standard of care in the management of patients with EC, with evidence suggesting improved perioperative outcomes. However, proper patient selection and surgical planning are crucial to avoid unplanned conversion to open surgery. Factors such as uterine size, prior abdominal surgeries, surgical complexity, disease extent, and histologic types can serve as reliable predictors for the conversion to open surgery. Overall, a tailored approach to surgical management, taking into consideration individual patient characteristics, is essential to optimize outcomes in patients with EC.

## Data Availability

The source of the data is the participating hospital(s) that submitted data to the SGO. “The Society of Gynecologic Oncology and the hospitals participating in the SGO COR are the source of the data used herein; they have not verified and are not responsible for the statistical validity of the data analysis or the conclusions derived by the authors”.
